# A Meta-Analysis to Evaluate Implant Survival and Benefits of the Use of Dual Mobility Constructs in Total Hip Replacement Following Hip Fracture

**DOI:** 10.7759/cureus.58755

**Published:** 2024-04-22

**Authors:** Hamish Macdonald, Andrew Gardner, Adrian Sayers, Jon Evans, Michael R Whitehouse

**Affiliations:** 1 Trauma and Orthopaedics, Musgrove Park Hospital, Taunton, GBR; 2 Trauma and Orthopaedics, University of Bristol, Bristol, GBR; 3 Bristol Medical School, University of Bristol, Bristol, GBR; 4 Musculoskeletal Research Unit, University of Bristol, Bristol, GBR

**Keywords:** intracapsular femoral neck fractures, dual mobility total hip arthroplasty, acute total hip replacement, total hip replacement (thr), geriatric hip fracture

## Abstract

Total hip replacement (THR) is commonly performed to treat hip fractures. Dual-mobility constructs (DMCs) are increasingly used for this indication. The aim of this study was to use evidence synthesis techniques to estimate net all-cause construct survival for THR with DMC performed for hip fracture. Additionally, we aimed to investigate and describe differences in all-cause construct survival (if present) between THRs performed with DMC (DMC-THR) or with a conventional bearing construct following hip fracture.

We performed a systematic review and meta-analysis of published studies (including joint registries), including DMC-THR for hip fracture which provided Kaplan-Meier (KM) survival estimates. The primary outcome was all-cause construct survival over time. The study was prospectively registered on PROSPERO (CRD42020173117).

A total of 557 papers and 17 registry reports were identified. Six studies (four registry reports, one matched-pair cohort study utilising joint registry data, and one single-institution case series) met the inclusion criteria, including 17,370 DMC THRs and 167,377 conventional THRs. Five-year KM survival estimates (95% confidence intervals) were similar at 95.4% (94.9 to 95.8%) for DMC-THR and 96.2% (96.0 to 96.4%) for conventional THR. The relative risk of revision for DMC-THR at five years was 1.21 (1.05 to 1.41). These results suggest that DMC-THR has a lower all-cause survival than conventional THR following hip fracture. This analysis does not support the routine use of DMC-THR over conventional bearing THR.

## Introduction and background

Approximately 65,000 hip fractures occur annually in the United Kingdom [1]. The global annual burden of hip fractures is predicted to rise to between four and six million by 2050 [2,3]. In the United Kingdom, the National Institute for Health and Care Excellence (NICE) issues national treatment guidelines; they recommend consideration of total hip replacement (THR) for patients with a displaced intracapsular fracture, who were independently mobile outdoors prior to fracture, are medically fit for the procedure, and are expected to remain independent for at least two years [4]. There is significant variation in the use of THR in this context [5]. In 2019, the last year for which figures were unaffected by the coronavirus disease 2019 (COVID-19) pandemic, 5,140 THRs performed for hip fractures were recorded on the National Joint Registry (NJR) [6]. Studies examining the relative benefits of THR and hemiarthroplasty for intracapsular hip fractures have concluded that THR is associated with improved long-term function, but increased risk of dislocation [7,8]. A recent randomised controlled trial comparing THR to hemiarthroplasty in patients over 50 years of age with displaced intracapsular hip fractures did not show a clinically significant difference in patient-reported outcome measures (PROMS) scores up to two years post-operatively with the use of THR as opposed to hemiarthroplasty [9]. Despite this, longer-term outcomes assessed by PROMS and revision estimated from randomised trials are unknown. Previous studies have reported a higher relative risk of dislocation of 1.5 to six for THR following fracture as opposed to elective surgery [10,11]. Methods to reduce the increased relative risk of dislocation are key if we wish to give patients the benefit of improved long-term PROMS following surgery for hip fracture.

The dual-mobility construct (DMC) was developed by Gilles Bousquet in the 1970s [12]. The aim was to combine the benefits of Charnley’s low-friction arthroplasty (low torque and hence reduced acetabular loosening) and McKee’s large head arthroplasty (increased range before impingement, increased jump distance, and hence lower chance of dislocation). Survival rates for DMC of 93% to 95% at 10 years [13,14] and 74% at 22 years [15] for primary elective THR have been reported (which should be viewed in the context of NICE guidelines recommending the use of implants with >95% 10-year survival [16]). For revision, THR 96% survival at eight years has been observed [17]. Concerns have been raised regarding rates of cup loosening [18], and the 2022 NJR report showed an increased incidence of revision, particularly for infection and periprosthetic fracture, with DMC as opposed to conventional constructs (for all primary THRs) [19]. Intra-prosthetic dislocation (IPD), a specific complication of DMCs, where the femoral head dislocates from within the mobile liner, has been reported to occur at rates of up to 4% [20].

Previous reviews have examined the use of DMCs for all THRs, including primary (elective and trauma) surgery and revisions [21-28], or revisions alone [29]. One previous review analysed the outcome of 554 DMC-THRs following a hip fracture as part of a broader review of DMCs for THR (including elective and traumatic indications) [26], whilst another investigated the use of DMC-THR for hip fracture compared to conventional THR and hemiarthroplasty [30], with 4,650 DMC-THRs included. A more recent individual-patient-data registry meta-analysis concluded that there was no significant difference in revision rates between DMC-THR and conventional THR [31]. We aimed to synthesise an all-cause construct survival estimate for DMC-THR performed for hip fracture utilising all available contemporary evidence, including registry reports and peer-reviewed literature, and to interpret this within the context of the survival of conventional THRs performed for hip fracture.

## Review

Methods

Search Strategy

This review was prospectively registered on PROSPERO (CRD42020173117). A systematic literature search was performed on June 28th, 2020, and repeated on July 15th, 2023, searching MEDLINE, EMBASE, Cochrane Central Register of Controlled Trials, and Web of Science. Reference lists of selected studies and previous reviews [21-28,30-32] were examined to identify relevant studies not selected by search criteria, and the process was repeated until no further studies were identified. All available national joint registry reports (Australia, Canada, Denmark, Finland, Germany, Ireland, Italy, the Netherlands New Zealand, Norway, Portugal, Romania, Scotland, Slovakia, Sweden, England, Wales, Northern Ireland, the Isle of Man and States of Guernsey) were examined for relevant data.

Determination of Study Eligibility

Following the exclusion of duplicates, an initial title screen was performed by the first author to exclude obviously irrelevant results. A review of abstracts and then full texts was performed independently by two authors. Disagreements were resolved by discussion between the reviewing authors. We included any case series (of at least five patients), cohort study, registry report or randomised control study with at least one group consisting entirely of patients receiving DMC-THR for hip fracture and which reported all-cause implant survival by means of a Kaplan-Meier (KM) estimate. Case series with fewer than five patients were considered likely to have an unacceptably high risk of bias given the frequency of hip fractures - most units will experience a sufficiently high case volume that a series of less than five raises the suspicion of reporting bias. Studies meeting the inclusion criteria were only excluded if they did not include an English language summary if they were a conference abstract only (as they were unlikely to contain the required information for meta-analysis), or if patients were also reported in another included paper or registry, in which case the larger study (by number of DMC-THR) was included. Studies that did not include a KM estimate were excluded, as the KM estimate is considered the most appropriate estimate of the true net failure rate [33].

Data Extraction

A study-specific data extraction form was created using Excel (Microsoft, Redmond, Washington, USA). Data were extracted by the first author and checked by a second researcher. KM estimates and their confidence intervals were extracted. For comparative studies, data were extracted for patients receiving DMC and conventional THR separately. When KM estimates and/or their confidence intervals were only available in graphical form, they were extracted using DigitizeIt v2.5 (I. Bormann, Braunschweig, Germany). 

Analysis

STATA v.16.1 (StataCorp, College Station, Texas, USA) was used for all statistical analyses. The primary outcome was all-cause construct survival. Planned secondary outcomes included dislocation, mortality, functional outcomes, patient-reported outcome measures, and cause-specific revision, but due to the lack of sufficient appropriate data, it was not possible to evaluate these outcomes. Pooled survival estimates for DMC and conventional THRs based on KM estimates and weighted according to standard error were generated, along with 95% confidence intervals, using the same method as used in similar reviews [34]. These were used to generate a survival curve for each construct type. Direct statistical comparison of different cohorts was not conducted as these would be susceptible to selection bias and confounding by indication.

Risk of Bias Assessment

Bias was assessed using the non-summative method as previously described by Wylde et al. [35] as this was developed specifically for joint replacement, rather than the more generic MINORS [36], which is less specific and contains multiple subjective criteria. This method is a tool based on whether studies are multicentre, whether they include consecutive patients, whether they have <20% loss to follow-up, and whether they utilise multivariate analysis.

Results

Literature Search and Baseline Characteristics

**Figure 1:**
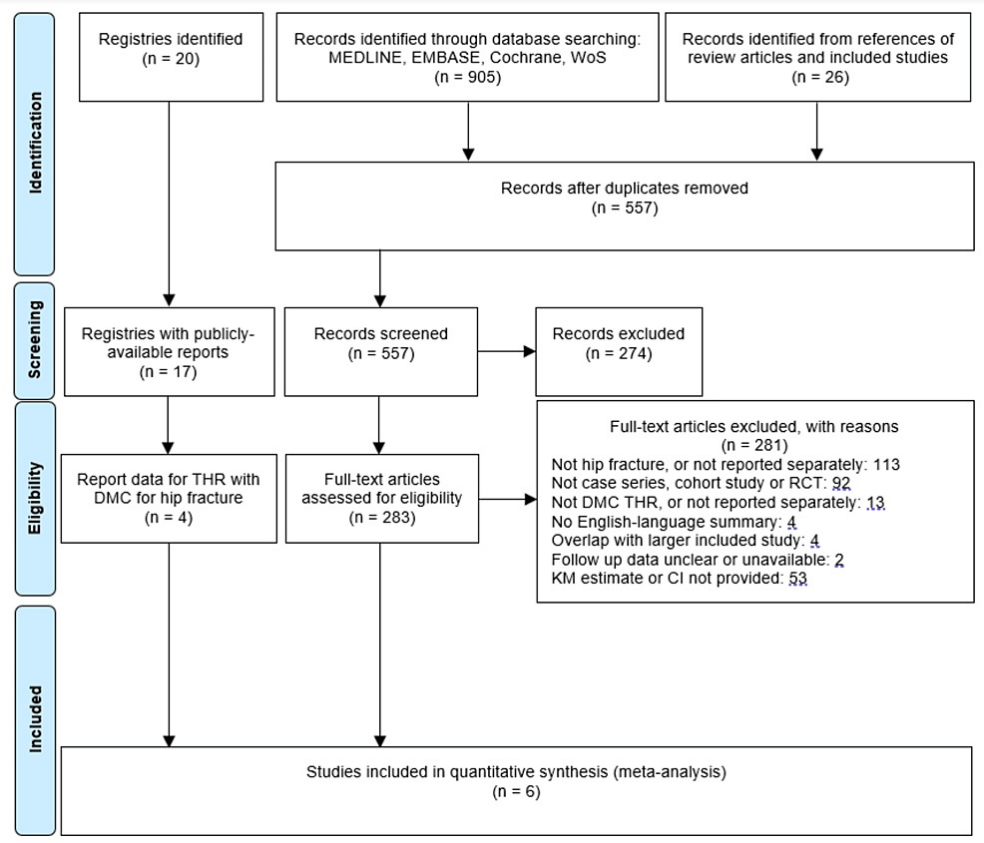
PRISMA flow diagram PRISMA - Preferred Reporting Items for Systematic Reviews and Meta-Analyses; WoS - Web of Science; RCT - Randomised Controlled Study; KM - Kaplan-Meier.

A total of 905 papers were identified from literature searches and an additional 26 from the review of references. Following duplicate removal, 557 records were screened, of which 274 were excluded based on title, leaving 283 full-text articles for review. Two hundred and eighty-one articles were excluded, leaving two for inclusion in the meta-analysis [37,38]. Seventeen published national joint registry reports were available, of which four [19,39-41] provided relevant survival information and so were included in quantitative analysis. Agreement on inclusion was reached in all cases. A Preferred Reporting Items for Systematic Reviews and Meta-Analyses (PRISMA) flow diagram is provided (Figure 1).

Table 1 shows available summary data for included studies and registries. In total, 17,370 DMC THRs and 167,377 conventional THRs were included in the meta-analysis.

**Table 1:** Baseline characteristics of included studies and joint registries AOANJR - Australian Orthopaedic Association National Joint Registry; GAR - German Arthroplasty Registry; SAR - Swedish Arthroplasty Registry; UK NJR - The National Joint Registry (covering England, Wales, Northern Ireland, the Isle of Man and Guernsey); DMC-THR - Dual-mobility construct-Total hip replacement * mean (standard deviation); † median (IQR). Blank cells indicate data not provided.

	n			Age			Male sex		
	DMC-THR	Conventional THR	Total	DMC-THR	Conventional THR	Total	DMC-THR	Conventional THR	Total
Jobory et al. 2019 [37]	4,520	4,520	9,040	77 (10.8)*	75 (10.9)*	76 (10.0)*	30%	32%	31%
Uriarte et al. 2021 [38]	105	0	105	76 (7.6)*			24%		
AOANJR 2022 [39]	4,128	20,673	24,801						
GAR 2022 [40]	1295	22,225	23,520	81 (74-85)†	76 (67-80)†		31%	30%	
SAR 2022 [41]	4,708	72,565	77,273			81 (9.33)*			36%
UK NJR 2022 [19]	2,614	47,394	50,008			73 (66-79)†			28%

Risk of Bias Assessment

Table 2 shows the risk-of-bias assessment for the included studies and registries. Most of the quantitative analysis data comes from multicentre studies, with low loss-to-follow-up rates and either consecutive patients or a justified reason (matched-pair analysis) for non-consecutive patients.

**Table 2:** Risk of bias assessment for included studies and joint registries The Jobory et al. [37] paper was considered justified in its use of non-consecutive patients as it was a matched-pair case-control study. AOANJR - Australian Orthopaedic Association National Joint Registry; GAR - German Arthroplasty Registry; SAR - Swedish Arthroplasty Registry; UK NJR - The National Joint Registry (covering England, Wales, Northern Ireland, the Isle of Man and Guernsey).

	Multicentre	Consecutive patients	<20% Loss to follow up	Multivariate analysis
Jobory et al. 2019 [37]	Yes	No (justified)	Yes	Yes
Uriarte et al. 2021 [38]	No	Yes	Yes	Yes
AOANJR 2022 [39]	Yes	Yes	Yes	Yes
GAR 2022 [40]	Yes	Yes	Yes	No
SAR 2022 [41]	Yes	Yes	Yes	No
UK NJR 2022 [19]	Yes	Yes	Yes	No

All-Cause Construct Survival

**Figure 2:**
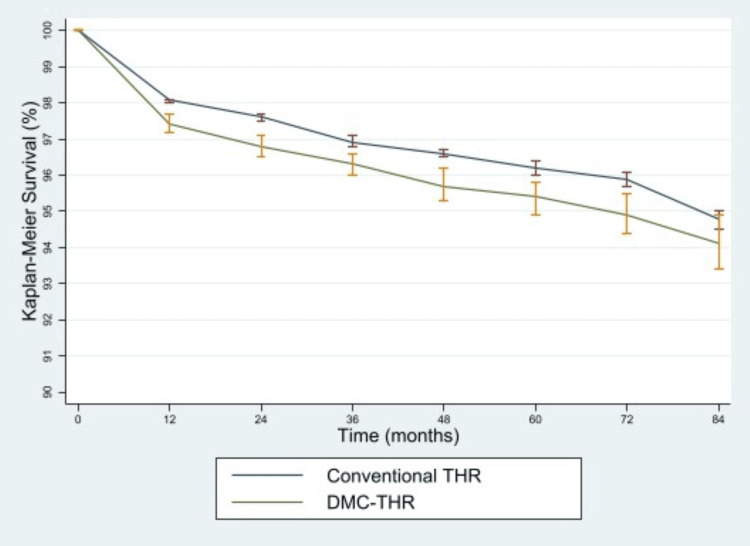
KM survival curves for conventional and DMC-THR KM - Kaplan-Meier; DMC - Dual-mobility constructs; THR - Total hip replacement.

Figure 2 shows KM estimates and 95% confidence intervals for DMC and conventional THRs for hip fracture. At all time points, all-cause construct survival was higher for DMCTHR than for conventional THR. The 95% confidence intervals for the survival estimate did not overlap until seven years. Overall five-year KM survival estimates for total hip replacement performed for hip fracture were 95.4% (94.9% to 95.8%) for DMC THR and 96.2% (96.0% to 96.4%) for conventional THR. This gives a relative risk of revision for DMC-THR of 1.21 (1.05 to 1.41) compared to conventional THR.

Discussion

Pooled Kaplan-Meier survival estimates suggest a slightly higher revision rate for DMC-THR than for conventional THR following hip fracture. Although the absolute increase in revision is low, this still equates to a 20% increase in revision risk when compared to conventional THR. This contrasts with the previous meta-analysis, which was based on studies with a generally high risk of bias and which did not report KM estimates either for all-cause implant survival, or risk of dislocation [30] and which suggested a significant benefit to the use of DMC-THR. The most recent analysis, based entirely on registry data, concluded that there was a slightly higher revision rate for DMC-THR, but with overlapping confidence intervals [31]. Other than one pilot study [42] (which concluded that a full-scale randomised trial was not feasible), no published randomised trials in this area were identified. A randomised trial of hip fracture patients examining the clinical and cost-effectiveness of DMC and conventional constructs is currently underway [43]; their results will add significantly to this topic.

This is the first study to meta-analyse all published survival data on DMC and conventional THR for hip fracture, including both peer-reviewed and registry data. As such, we have included more DMC-THRs than in previous studies. Even though 2,790 DMC-THRs were excluded due to the lack of KM estimates, this study still included 17,370 DMC-THRs performed for hip fractures. Registries form a vital part of contemporary joint replacement literature and have a high capture rate - the NJR is compulsory, with data capture rates of 96.7% for primary hip replacement, whilst the AOANJRR reports >95% compliance when compared with health service records. 2019 was the first calendar year for which the NJR reported outcomes for DMC-THR, significantly expanding the published body of literature.

By pre-defining inclusion and exclusion criteria and registering the search strategy and planned analysis, bias from selective inclusion or interpretation was minimised, and the best estimate of all-cause construct survival for DMC-THR was provided by utilising KM estimates.

This study does have limitations. It is not possible to draw conclusions regarding the reason for the difference in survival seen, as there was no available data regarding the reason for revision in the registry reports. Previously published work, including studies excluded from this analysis due to lack of KM estimates, has consistently shown a reduced revision rate for dislocation for DMC-THRs [21,30], whilst a review of risk factors for dislocation following elective primary THR found DMC to be protective against dislocation [44]. It may be that there is selection bias affecting the DMC-THR group, whereby patients at higher risk of dislocation - who may be older, frailer, more comorbid, and more prone to recurrent falls, receive this implant rather than a conventional articulation, thus causing confounding by indication. The recent study by Farey et. al [31] did indeed find that DMC-THR patients were older and more comorbid than those receiving a conventional THR. These risk factors are not specific to dislocation; however, they may also place the patient at higher risk of revision for alternative causes. This selection bias would tend to lead to poorer outcomes for DMC-THR than might be seen otherwise. Potentially driving results in the other direction, however, is the fact that if patients selected to receive, DMC-THR are indeed at inherently higher risk of complications, then these complications might be more likely to be managed non-operatively or through non-revision operations not captured on joint registries. The NJR has started to collect data on such reoperations, which will inform the debate further.

Alternatively, specific characteristics of DMC-THR might predispose it to higher revision rates. The dual articulations, particularly of the mobile liner with the acetabular component, are an additional source of polyethylene wear particles, which are a driver of osteolysis and implant loosening [45]. DMC-THRs are also at risk of intra-prosthetic dislocation, which is a complication specific to this variety of THR [20]. Finally, improved resistance to dislocation may have a direct negative effect on the risk of periprosthetic fracture: if a torsional force is applied through the THR (for example during a fall), that cannot be dissipated by means of dislocation (due to the increased stability of DMC-THR), this may instead lead to a periprosthetic fracture. This has not been formally examined but could be investigated by means of a cadaveric study.

A final limitation is that all-cause implant survival may not be the outcome of most interest to patients [46]. If the added stable range of a DMC-THR facilitates a less restricted lifestyle [47], then it should be established whether patients consider this an important factor, which would require discussion with patient engagement or priority-setting groups. 

## Conclusions

Given the incidence of hip fracture and its clinical and financial implications for patients and health services, the surgery provided must comply with high-quality evidence-based recommendations. This largest study of its type suggests that DMC-THR may have a higher revision rate than conventional bearings. The additional cost of DMC implants in revision surgery has been estimated at £1,100. If this additional cost is to be justified, then high quality evidence should exist for clinical and cost-effective benefit. This research, therefore, concludes that from the available literature, we have not observed any justification for the increasing use of DMC-THR to treat hip fractures. DMC components are more expensive than conventional implants and may be associated with increased rates of revision. Surgeons should instead use conventional implants with which they may be more familiar.
